# Handling Real-World Context Awareness, Uncertainty and Vagueness in Real-Time Human Activity Tracking and Recognition with a Fuzzy Ontology-Based Hybrid Method

**DOI:** 10.3390/s141018131

**Published:** 2014-09-29

**Authors:** Natalia Díaz-Rodríguez, Olmo León Cadahía, Manuel Pegalajar Cuéllar, Johan Lilius, Miguel Delgado Calvo-Flores

**Affiliations:** 1 Åbo Akademi University, Department of Information Technologies, Turku Centre for Computer Science (TUCS) - Joukahainengatan, 3-5, Turku FIN-20520, Finland; 2 University of Granada, Department of Computer Science and Artificial Intelligence, E.T.S.I. Informática y de Telecomunicación -C/. Periodista Daniel Saucedo Aranda s.n., Granada 18071, Spain

**Keywords:** 3D depth sensors, activity recognition, fuzzy ontology, context awareness, ambient intelligence, semantic web, uncertainty, vagueness, hybrid systems

## Abstract

Human activity recognition is a key task in ambient intelligence applications to achieve proper ambient assisted living. There has been remarkable progress in this domain, but some challenges still remain to obtain robust methods. Our goal in this work is to provide a system that allows the modeling and recognition of a set of complex activities in real life scenarios involving interaction with the environment. The proposed framework is a hybrid model that comprises two main modules: a low level sub-activity recognizer, based on data-driven methods, and a high-level activity recognizer, implemented with a fuzzy ontology to include the semantic interpretation of actions performed by users. The fuzzy ontology is fed by the sub-activities recognized by the low level data-driven component and provides fuzzy ontological reasoning to recognize both the activities and their influence in the environment with semantics. An additional benefit of the approach is the ability to handle vagueness and uncertainty in the knowledge-based module, which substantially outperforms the treatment of incomplete and/or imprecise data with respect to classic crisp ontologies. We validate these advantages with the public CAD-120 dataset (Cornell Activity Dataset), achieving an accuracy of 90.1% and 91.07% for low-level and high-level activities, respectively. This entails an improvement over fully data-driven or ontology-based approaches.

## 1. Introduction

Human activity recognition (AR) is a complex and key aspect in the development of ambient intelligence (AmI) systems. Different techniques have been developed for activity and user modeling, and they may be classified as data-driven [1] and knowledge-based [2] approaches. The methods in the former category aim at providing models for handling human behavior-specific features using statistical and machine learning techniques. The strengths of these models are their ability to handle noise, uncertainty or incomplete sensor data [3], and they have proven to be accurate in different domains where semantics are not key. However, the need for training data, fast adaptation to a habit or environmental changes and the experiments required to achieve a suitable performance are limitations in dynamic environments and situations where context-aware data prevail. Since it is usual to deal with a continuous incrementation/removal/change of sensors due to user mobility, sensor failures, lack of battery and other factors, the re-training of data-driven techniques may become a drawback of these systems. Furthermore, data-driven algorithms do not offer abstract reasoning mechanisms that allow the inference of the meaning of actions according to their semantics [4].

On the other hand, knowledge-based techniques have been applied in pervasive computing environments to improve interoperability and adaptation to different context situations [4–10]. Usually, context data sources are dynamic, are continuously changing, depend on the environment and are not always mobile, known, nor taken into account in advance [5,11]. For this reason, these methods show advantages with respect to data-driven models due to the inclusion of context management tools, such as common sense knowledge encoding. Further features of knowledge-based techniques that are interesting for human activity representation are the possibility of providing both the environment and the user with semantics to aid in the context definition process, facilitate the definition and comprehension of human behaviors (e.g., machine readability and easy interpretability) and, consequently, ease the development of new learning and recognition models able to better understand the meaning of human actions and execute logic reasoning about future needs, situations or actions. All of these procedures can be implemented considering the context information where the activity is being performed. Examples of knowledge-based techniques encompass logic-based approaches [12,13], rule-based systems [14] and ontological models [7].

Although the most widely used tool to integrate semantics into activity recognition systems are crisp ontologies [4], they present some limitations when they are applied to activity recognition. For instance, they require good knowledge engineering skills to model the domain, and the Web Ontology Language (OWL) Description Logic (DL) does not allow interval (*i.e.*, overlapping) temporal reasoning; and they cannot deal with uncertainty [4]. However, a fuzzy formulation of an ontology for human behavior recognition may help to overcome these limitations, such as missing sensor readings, input from parallel or interleaved activities being performed at the same time or management of vagueness and incomplete data. Since the nature of human behavior is non-deterministic, we believe that a fuzzy ontology can deal with these phenomena inherently.

In this work, we use a fuzzy ontology [5] on top of a data-driven sub-activity recognition system to give support for semantic interpretation, logic reasoning and management of imprecision and uncertainty in activity recognition scenarios. Our rationale is that a sensor can provide readings with a certain degree of reliability or sample data only at specific times or under certain conditions; users may perform subtle changes in the way they perform their activities; the execution of an activity may be detected with uncertainty or a satisfiability degree; and the performance of an atomic action (for example, to take a dish) depends on the goal or intention of the user. In this last case, semantic interpretation of human actions is key to achieving a suitable activity recognition. As a consequence, all of this information should be taken into account in the reasoning and recognition process.

Fuzzy ontologies have great advantages with respect to crisp ones [5,15]. They have been shown to be useful in domains such as reaching consensus for group decision making [16] or extending information queries to allow the search for incomplete results [17]. In the domain of activity recognition, they allow one to define that an activity, *i.e.*, *CoffeeBreak*, is recognized, taking into account the relevance of its involved sub-activities using weighted aggregation (e.g., 0.3 *TakeMug*, 0.3 *TakeCoffeePan*, 0.4 *TakeMilk*). Thus, when a sub-activity has been skipped due to an exception (e.g., milk running out) or a missing sensor reading, the activity can still be recognized with a lower degree of reliability. In contrast, the same activity, if it is formalized in a crisp ontology, could not be recognized if any of the exclusive sub-activities are missing.

In a previous work, we have demonstrated theoretically that fuzzy ontologies can be used to improve the accuracy in activity recognition scenarios with respect to crisp models [5]. In this paper, we extend the previous work [5] and create a hybrid fuzzy ontology/data-driven approach that embraces the benefits of both methodologies. On the one hand, a data-driven model is in charge of processing sensor data directly to recognize atomic human activities, *i.e.*, walk, take/release an object, sit down, *etc*. We call these activities atomic since they cannot be explained as a sequence of simpler activities and are always performed in the same way. Thus, it is not necessary to perform re-training of the data-driven model for its adaptation to new instances of the same activity. On the other hand, these atomic actions (sub-activities) are input to the knowledge-based system composed by the fuzzy ontology, which provides the results from the data-driven module with semantics and performs ontology reasoning to infer more abstract and complex activities, *i.e.*, cooking, washing dishes, *etc.* By using a fuzzy ontology, the benefits of expressibility and looseness in the activity models allow a higher tolerance to the inherent uncertainty and vagueness of the problem. In the experimental section, we validate our approach over a benchmark dataset (Cornell Activity Dataset, CAD-120). In summary, our proposal's contributions can be enumerated as:

- The ability to handle imprecise, vague, uncertain or incomplete data in ontological AR, such as missing sensor readings, in real-time environments. This model is validated with a public dataset of complex activities containing 3D-depth user and objects position data (*i*.*e*., it does not require using wearable sensors). This dataset includes activities in a continuous video data stream. Then, a user tracking system is required.
- A method that simplifies complexity in the training phase. In the case of the new addition/removal/replacement of input data, instead of re-training, it is only required to modify the affected activity rule in the fuzzy ontology, since the activities and their relationships can be modeled as common sense rules.
- A hybrid system that integrates the benefits of machine learning with the ability to include semantics and improve the model interpretation, typical of using knowledge engineering methods.
- A two-stage activity recognition framework, based on the detection of atomic and abstract activities using the hybrid data-driven/knowledge-based methodologies. This system leads to obtaining results with statistically significant improvements with respect to previous approaches, as we show in the experimental section.
- The proposal can be implemented as a real-time recognition system that can monitor and recognize activities (e.g., to assist independent elders). In our experiments, we achieved an average time of 0.56 s for the recognition of an activity, on average.

The remainder of the manuscript is organized as follows: the following section describes related work on existing approaches employing data-driven and knowledge-based human activity recognition models, where the advantages and limitations of these methods are also discussed. The proposal of the hybrid activity recognition model is presented in Section 3. After that, Section 4 details how uncertainty is tackled in the fuzzy ontology, and Section 5 shows the experiments for the validation. Finally, Section 6 discusses the results, and conclusions and future work are summarized in Section 7.

## 2. Related Work

In order to model human activity and behavior in AmI, the context needs to be modeled. With respect to other context models, such as key-value models, object oriented or logic-based models [11], ontology-based context modeling excels with regards to simplicity, flexibility, extensibility, generality, expressiveness and automatic code generation [9]. On the other hand, few solutions combine the benefits and robustness of traditional statistical data-driven methods with ontological knowledge-based approaches for AR. In this section, we discuss the latest trends in this area.

### 2.1. Data-Driven Approaches for Human Activity Recognition Based on Video Signals

There is much literature dealing with the human activity recognition problem, covering fields such as computer vision, pattern recognition, signal processing, machine learning, *etc*. [18]. Human activity recognition has been a widely studied area in computer vision for decades, as the use of video-cameras may provide all of the necessary information from the scene. However, additional complexity is introduced if we are looking for a system capable of understanding what is happening in the images from the video stream. Further limitations of these techniques are occlusions, cluttered background, shadows, varying illuminations and viewpoint changes. At the moment, there are few proposals that are able to learn and detect complex behaviors, such as ADLs, using video data, although in the last few years, there has been an increasing effort in the field of automatic gesture recognition as a first step.

Depth cameras appeared on the market in the last few years and are useful for overcoming some of the limitations of raw video cameras, such as shadows, viewpoint changes and body detection. The release and popularity of the Microsoft Kinect provides RGB image and depth image streams [19]. Although targeted initially for the home entertainment market with the Xbox console, the Kinect has received increasing interest from the vision and robotics community due to its great potential [20]. A good example of its possible application is detecting the presence of people in a scene. In the work of Salas [21], a strategy that combines color and depth images (obtained by the Kinect device) by histograms of oriented gradients (HOG) to detect people in indoor environments is presented. This strategy greatly improves previous work in this field, obtaining high accuracy (up to 34,981 targets could be detected in an image). This people detector is efficient and accurate from the computational point of view and was validated in a pilot phase. Another work that tries to solve the activity recognition problem can be found in [22], where a method to quickly and accurately estimate 3D positions of the user's skeleton joints from a single depth image from the Kinect was proposed. With this kind of information, we can address the problem of human action recognition in a simpler way compared to the use of classic RGB images.

In the last two years, there has been a wide number of approaches for human activity recognition using depth cameras. For instance, in proposals, such as [23,24], the authors develop a technique that integrates EigenJoints [25] to find movement features. The learning and recognition is executed by means of naive Bayes nearest neighbor (NBNN) and SVM. For the experimental phase, they use a dataset called MSRAction 3D, containing 20 actions that were chosen in the context of interacting with game consoles. These authors achieve a high classification rate, ranging from 71% to 97%, depending on different activities taken from the dataset. Despite these great advances, the actions performed in this dataset do not consider interaction with the environment and are simple actions (assuming simple as atomic or low abstraction level activities), so that they cannot be used to evaluate AAL scenarios.

Another human action recognition framework is [26], which includes different stages or modules. First, the 3D skeleton data of the user is captured and processed by means of histograms of the 3D joint locations. After that, linear discriminant analysis (LDA) is calculated, and the data are quantized. Then, the learning and classification of the activities are carried out with hidden Markov models. The selected actions for the dataset in this reference were: walk, sit down, stand up, pick up, carry, throw, push, pull, wave and clap hands. Different tests are implemented for the experimentation, achieving recognition rates between 79% and 97%. The accuracy and detection success of this method are also promising, although the design for the recognition method does not allow one to include features to model interactions between the user and the environment. The objectives of [26] and our proposal differ substantially, since ours also includes processing and recognition of the user interaction with objects. Since ground assumptions are not the same for the problem statement in this work and [26], we cannot use the same dataset for comparison. Instead, we selected another benchmark dataset provided in [27] that also includes user-object interaction.

In [27], a complete, rich (continuous and discrete) dataset (called CAD-120) is provided together with a method that produces promising recognition accuracy, based on RGB-D video. Human activities and object affordances are modeled as a Markov random field, and the learning is done by a structural support vector machine (SSVM). The authors tested their proposal on this dataset comprising 120 activity videos collected from four subjects and obtained an accuracy of 79.4% for object affordances, 63.4% for sub-activity detection and 75.0% for high-level activity labeling. Finally, they demonstrated the use of such descriptive labeling in performing assistive tasks by a PR2robot. We have tested our proposal with this dataset, as it is the most complete and challenging one that we have found with respect to ADL scenarios, user-environment interaction and that is based on RGB-D video.

An interesting, but different, data-driven approach is in [28], where relevance weights from the web are obtained from websites, such as wikihow and ehow.com, in order to mine object terms and relevance weights from comprehensive instructions for daily activities. It focuses on solving the activity boundary detection problem or trace segmentation problem when mining new activity patterns. Uncertainty is handled in this case using data-driven crawling-like methods and not ontologies and fuzzy logic (as we will do for automatic knowledge inference). Their unsupervised approach for activity recognition and segmentation is based on object-use fingerprints and aims at avoiding the required labeling stage typical of activity recognition systems. This is an appropriate approach for future inclusion of new activities or activities where object usage traces are not known in advance. This procedure could speed up the modeling in the ontology for it to evolve and adapt to new human behaviors.

The works described previously have focused on trying to produce a high ratio of correct classification and detection, but they have not addressed the problem of real-time activity recognition, which might be key in applications and environments, such as the home, office or public spaces, among others. The adaptation of the proposed learning and recognition models to the requirement of real-time processing is not trivial, since the algorithm complexity and the big amount of data to be processed are limitations that are hard to overcome. Indeed, it is difficult to find in literature works that include time complexity information in their experiments for the problem we address. In the experimental section of this work, we will include information about computational cost and show that our model could be used in scenarios that require near real-time response. We complete these requirements with the presented framework in Section 3.

### 2.2. Ontological Knowledge-Based Approaches for Human Activity Recognition

An ontology is a formal specification of a shared conceptualization [29]. It offers a formalism to represent classes or concepts, individuals, relations, functions and attributes. As providers of a format for exchanging knowledge, they promote interoperability, knowledge reuse and information integration with automatic validation. Ontologies separate declarative and procedural knowledge and facilitate the modularity of the knowledge base (KB) [30]. Using ontologies in human activity recognition provides a number of advantages [31]: it supports incremental progressive activity recognition, state based modeling and a robust reasoning mechanism. Other benefits are the ability to discriminate the significance and urgency of activities through semantic descriptions, the support for course-grained and fine-grained activity inference and the possibility for data fusion and semantic reasoning, including activity learning, activity recognition and user assistance [5].

The Web Ontology Language (OWL) is based on the knowledge representation formalism of Description Logic (DL) [32], which models concepts, roles and individuals. In DL, the terminological box or TBox is the vocabulary used for defining concepts and roles within a domain, while all instances or named individuals conform assertions about a real-world domain in the ABox, which is the assertional box. While the TBox contains sentences describing concept hierarchies (*i*.*e*., relations between concepts), the ABox contains ground sentences stating where in the hierarchy individuals belong. Statements in the TBox and ABox can be interpreted with rules and axioms in DL to enable reasoning and inference, including satisfiability, subsumption, equivalence, disjointness and consistency. DL reasoning supports decidability, completeness and soundness in polynomial time complexity for an inexpressive DL and in exponential time complexity for expressive DLs [32].

Few approaches to human activity recognition have based their functioning on semantic approaches for automatic reasoning, such as ontologies. An in-depth survey of ontologies used in context modeling for human activity representation may be found in [4]. Ontologies have been shown to be useful in hybrid approaches for context reasoning, together with computer vision research integrating 2D scene tracking with ontological layers to avoid issues that make classical object tracking procedures fail in complex scenarios [33]. However, in our case, we take advantage of more precise 3D-Depth sensors and provide automatic treatment of the imprecision sources.

Other recent works that show that hybrid approaches are promising are, for instance, [34], where they model spatio-temporal scenarios with non-ontological fuzzy semantics, and [35], a concurrent activity recognition KCAR(crisp) ontological approach focused on concurrent multi-user activity recognition. However, the validation is done with a dataset where activities are discriminable by location characterization and where all data are discretely labeled (from different, but mainly positioning sensors). In our framework, we tackle both continuous and discrete streams of data.

Anther recent approach is [36], where ontological reasoning is used for non-concurrent real-time continuous recognition of fine and coarse-grained single-user activities. However, although it includes partial uncertainty (handling simulated faulty-sensors and changes in objects used), it does not combine domain knowledge with data-driven approaches nor, does it tackle a continuous input dataset. A 94.44% average activity recognition rate was achieved with an average recognition runtime of 2.5 seconds.

The proposal in [37] describes a probabilistic Markov chain analysis to discover representative activities through activity signatures that serve to generate complex activity definitions. They achieve high accuracy for concurrent and interleaved activities. Despite using a hybrid and unifying theoretical framework, exploiting both domain knowledge and data-driven observations based on context-driven activity theory (CDAT), the dataset employed is only discrete and synthetic. Furthermore, although it is a semantic approach, they do not use an ontology to fully exploit automatic knowledge inference nor uncertainty reasoning.

On the other hand, the work in [38] uses a knowledge-based method where an ADL ontology and a dynamic sensor data segmentation method [39], based on shrinking and expanding the time window, produce high accuracy recognition of daily activities. Their approach provides very good *ad hoc* results under a synthetic dataset; however, they do not provide results under realistic scenarios, which are always more complex and have an inherent component of uncertainty. On the other hand, this approach does not consider movement tracking for sub-activities and is evaluated with a discrete input stream as a whole, which does not always occur in practice, due to the heterogeneity of sensors for data acquisition.

A common lacking element found in existing hybrid and ontological AR systems is the support for modeling uncertain, vague and imprecise information [4,5], which is an inherent feature of activity recognition. Furthermore, there are also needs for hybrid AR systems that can tackle the problems of both low-level activity detection in real time, as well as the semantics, context-awareness and uncertainty typical of high-level activities. Uncertain or vague data should be used as a natural way to provide flexibility to the model, for its adaptation to real-life situations.

Our work is motivated by a lack of proposals tackling motion detection at the same time as figuring out the actual meaning of these activities, taking imprecise context into account. In addition, by considering context and a proper management of uncertainty, we will avoid the need for re-training the system when new input data or models change, according to the context. Therefore, our framework consists of two well-distinguished modules, described in the next two sections. The first modules will recognize low-level activities, with no specific meaning within a context, nor a complex activity definition associated. The second module will provide meaning to sequences of sub-activities to infer the significance or purpose of high-level complex activities.

## 3. A Hybrid Real-Time Activity Recognition Framework

**Figure 1.**
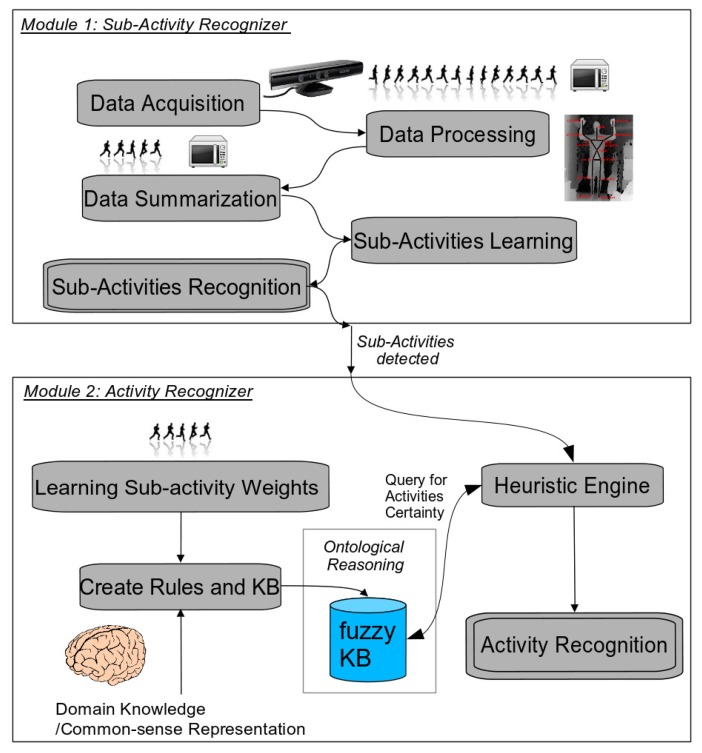
General diagram of the proposed hybrid framework.

The general proposed framework consists of two main modules (see Figure 1): the low level sub-activity recognizer and the high-level activity recognizer. Each module has been developed using different techniques: a data-driven approach for the first module and a knowledge-based context-aware one for the later. The first module detects actions or atomic activities that take input data directly from sensors. In our implementation, we have used dynamic time warping to learn and recognize these sub-activities, which is classified as a data-driven method. On the other hand, the second component lays on top of the previous one, in a superior level of abstraction. It gets input data from the first data-driven component (*i*.*e*., sub-activities) and executes ontological inference to provide, with semantics, both the activities and their influence in the environment. This component is thus knowledge-based, and we have used a fuzzy ontology to model high-level activities. In the following subsections, we provide the details to show how the whole system is able to detect and recognize complex human activities, such as ADLs, using an RGB-D sensor.

### 3.1. Phase 1: Data-Driven Modeling and Recognition of Low-Level Activities

In our activity recognition approach, we receive data from an RGB-D camera. By means of this sensor, we are able to extract data about the body postures of the users appearing in front of the sensor, as well as the 3D location of the objects in the scene. We focus on the time complexity of how to process the data received by this sensor efficiently and how to make the system capable of understanding the user's whole activity in real time, to get closer to real-time applications in daily life. The device selected for this task is the Microsoft's Kinect camera, which offers depth and RGB images of the scene.

Our goal is to create a data-driven framework that learns, detects and recognizes different events performed by users at their homes. Each one of these events or sub-activities will correspond to a sequence of images obtained by the camera, in this case a sequence of the user's body postures and the objects that take part in the sub-activity. The learned sub-activities will be used in the upper layer to detect and recognize more complex human activities, such as ADLs (involving one or more of these sub-activities). Once the data are acquired from the sensor, the sub-activity recognition framework comprises four additional steps:

- **Step 1: Initial processing of the 3D data provided by the camera.**
  **1.a: User's posture detection.** We obtain the user's body skeleton represented as a set of fifteen 3D points (head, neck, torso and left and right shoulders, elbows, hips, hands, knees and feet) through the camera and the middleware used. Afterwards, we process this data to represent the user's skeleton in a manner that is independent of the angle and distance to the camera. This representation will be the set of angles conformed by the user's body joints (angles between bones), plus the measured height value of the user's chest from the floor. In the end, the body posture on each frame received by the camera is represented by a set of eleven float values (ten angles plus the height).
  **1.b: Object detection and tracking.** The detection and tracking of objects in the scene must be carried out to infer and detect sub-activities involving their use. There already exist solutions to this problem as in [40], and we have used the same implementation as in [27]. The process consist of: (1) learning the features of a set of objects that can be placed in the scene; in this work, we acquired the objects included in the CAD-120 dataset and learned the RGB-D object dataset by Lai *et al.* [41]; (2) the 3D bounding boxes of the objects detected, with a score above a threshold, are obtained; and (3) the object tracking can be done using the particle filter tracker implementation provided by the PCL library (Pointclouds library http://pointclouds.org/documentation/). The result is a list of objects detected at every frame with a given 3D position of the centroid.
- **Step 2: Compressing the data time series.**
  In our problem, the time series data is the sequence of postures performed by the user and the object positions. We summarize the sequence of postures in order to work with a smaller amount of data. The method selected for this task has been the piecewise aggregate approximation (PAA) [42].
- **Step 3: Training the model for sub-activities learning.**
  The objective of this phase is to learn different sub-activities involving the use of objects by the user, for its later recognition. To this end, we use instance-based learning using the k-nearest neighbors algorithm, where we have an instance database and each instance is labeled with an activity. We select a subset of instances of the recorded sub-activities in a training dataset as template activities, so that new instances acquired from the sensor are compared to the templates. The distance measure to compare these time series instances is the algorithm, dynamic time warping (DTW) [43], which measures the similarity between two sub-activities and is invariant to their length, lags and speed. For instance, similarities in walking patterns could be detected using DTW, even if one person was walking faster than the other or if there were accelerations and decelerations during the course of an observation. The DTW time complexity is *O(m·n)*, where *m* and *n* are the length of the time series instances. We selected this technique for training and recognition of activities, because it provided the best results in both accuracy and time complexity in a preliminary experimentation. As an example, the work published in [44] describes a comparison of DTW with HMM, where we conclude that the use of the proposed framework with DTW results in a faster execution time and recognition accuracy.
- **Step 4: Sub-activities recognition.**
  Finally, once we have the different models trained, the system will be able to detect and recognize a new sub-activity sample performed by the user. The usual operation is that, after the depth sensor produces a new RGBD image sample, this is used as input for Steps 1–2 explained above and, then, compared within the different trained models of Step 3. Each one will return an output score, reflecting the similarity of the input sequence that conforms to the model. The instance in the database with the best membership score will be classified as the sub-activity being performed.

#### 3.1.1. Algorithm Settings and Feature Selection for Sub-Activity Recognition

This section describes the features used for recognition of sub-activities after the data are acquired from the RGB-D sensor in depth. In summary, we use two types of features: those for user detection and features for object affordance recognition.

1. **Skeleton features.** Once the framework has been implemented, the next step was to configure it before the experiments. Since we are using the CAD-120 dataset, some preliminary tests were done to see if the features selected initially were valid. We realized that the representation of the whole skeleton from the user was introducing some noise, as in most of the activities, only the upper body was used, and sometimes, the lower body was occluded. This problem is first motivated by the course of dimensionality, since motion is a multivariate time series. Therefore, finally, only the set of angles representing the upper body were selected as features. These are four angles, representing the user's arms independently from the camera position (see Table 1). This is actually an advantage, as we are reducing the amount of data to be computed, easing our aim of reaching real-time recognition. Future work will focus on making this feature selection automatically.
2. **Object features.** As shown in Table 1, 16 object features will be used as part of the global feature vector used for the DTW algorithm. The first restriction we applied is that the objects are only considered when the Euclidean distance between the object and any of the users' hands is less than 40 centimeters. While this happens, we keep track of the object by including its distance to the hand as part of the feature vector. This information is saved in different manners depending on the type of object and its identifier as follows:
  (a) if the object type cannot be found repeated within the same activity (e.g., there are not two microwaves), its distance will only be saved once in the appropriate cell (from 1 to 10, as there are 10 different object types) of the features vector related to the object type (see Table 1);
  (b) if the object type can be found repeated within the same activity (e.g., stacking several bowls), we use an object identifier, and its distance will be saved once in the appropriate cell (from 1 to 5, as the maximum number of repeated objects is five) of the feature vector related to the object identifier (see Table 1).
    Finally, we also want to represent the relationships between objects appearing in the global scene in another feature (see Table 1). To this end, we compute the sum of the Euclidean distances between all objects that take part in the activity, representing the objects movements. With these assumptions, we are able to reduce the amount of features to be computed in the next steps.
3. **PAA configuration.** The algorithm used to summarize the number of frames of each sub-activity sample was PAA. In this method, the time series are divided into k segments of equal length, and then, each segment is replaced with a constant value, which is the average value of the segment. Then, these average values are grouped in a vector, which represents the signature of the segment. In our experimentation, we have tested three different compression rates to compare which one performs better. The rates chosen were 2, 4 and 6, which reduce the frame samples to half, one quarter and one sixth of the total size, respectively. The best results were obtained with *PAA* = 2, and thus, this has been the configuration in the final experiments. If *X* = {*x*_1_,‥,*x_n_*} represents the time series, the *PAA* algorithm with compression rate *K* is represented by Equation (1), where *Y* = {*y*_1_,,‥,*y_m_*} represents the resulting time series.
  (1) $$y_{i}=\frac{\sum_{j=K*(i-1)+1}^{K*i}x_{j}}{K};\forall i\in \left\{1..\left\lfloor n/K\right\rfloor \right\}$$
4. **DTW configuration and recognition algorithm.** After the execution of the previous steps, we have a final feature vector of 20 dimensions in each component, as shown in Table 1. One vector represents a set of frames of the video stream. Since the features vector are of a different nature, they are normalized to the range [0,1], so that their weight does not unbalance the DTW algorithm operation. An in-depth description of the DTW algorithm may be found in [43].

**Algorithm 1:** Algorithm for sub-activity training using DTW.
**Input:** A finite set *T* = {*T*_1_, *T*_2_,…, *T_n_*} of training instances for the same sub-activity Each instance is a sequence of features vector (frames). *L_i_* is the length of each instance *T_i_***Data:** The *DTW*(‥) function takes two sequences *T_i_*, *T_j_* as input and calculates an optimal match of both, returning the calculated distance for them.**Result:**
*avgGlobal* is the average distance obtained after executing DTW for every pair of the training instances.*avgGlobal* ← 0**for**
*i* ← 1 ***to***
*n*
**do** **for**
*j* ← 1 ***to***
*n*
**do**  *distance* ← *DTW* (*T_i_*,*T_j_*)/(*L_i_* + *L_j_*  *avgGlobal* ← *avgGlobal* + *distance* **end****end***avgGlobal* ← *avgGlobal/n* * *n*


The system training comprises the creation of a set of instances labeled with their corresponding sub-activity Secondly, the DTW algorithm is applied for each pair of instances of the same activity in order to calculate the average distances between all of the training instances (*avgGlobal*; see Algorithm 1), which represents the average variation between different instances that the system may encounter during the recognition. The procedure *DTW* (*T_i_*,*T_j_*) in Algorithm 1 returns the cost of the optimal path that matches *T_i_* and *T_j_* [43], and this value is normalized using the sum of the lengths of the instances being compared, to avoid that this value depends on the lengths of the instances. This process is done separately for each sub-activity to be trained.

Once the system is trained, the recognition of a new sample can be carried out following Algorithm 2. This algorithm is applied for each sub-activity and returns an output score that measures the similarity between the new sub-activity acquired from the sensor data and the templates in the training set. As shown in Algorithm 2, we calculate the minimum distance between the new activity being recognized and the instances of each activity in the training set (*minDistance*), together with the the average distance to the training instances of each activity (*average*). Finally, a score in the range [0,1] (Equation (2)) is calculated to obtain the similarity between the sample and the activity training templates. This score is composed of two parts: firstly, the distance between the new sample and the nearest template in the training set for each activity, normalized to [0,1]; secondly, the difference between the average distance between training templates calculated in Algorithm 1 and the average distance between the new sample and the training set. The higher the value of the score is, the more similar the sample is to the compared activity templates. The parameter *α* is used to state the relative relevance that should be given to the minimum distance and the difference of average distances during the recognition stage. In our experiments, we found that the value *α* = 1/7 provided us with the best experimental results.

(2) score←max{NormalizeDistance(minDistance)−α|avgGlobal−average|,0}

**Algorithm 2:** Algorithm for sub-activities recognition using DTW.
**Input:** The *newSequence* is the sequence to be recognized. *L_newSequence_* is the length of *newSequence***Data:** A finite set *T* = {*T*_1_, *T*_2_,…, *T_n_*} of training instances for the same activity. Each instance is a sequence of features vector (frames). *avgGlobal* is the average distance obtained after the training stage. *L_i_* is the length of each instance T_i_. The function *NormalizeDistance*(‥) normalizes the input distance to a value between 0 and 1.**Result:** A score for the *newSequence*, is a float value between 0 and 1, 1 being the maximum score, related to the training set.*average* ← 0*minDistance* ← ∞**for**
*i* ← 1 ***to***
*n*
**do** *distance* ← *DTW* (*T_i_*, *new Sequence*)/(*L_newSequence_* + *L_i_*) **if**
*distance < minDistance*
**then**  *minDistance* ← *distance* **end** *average* ← *average* + *distance***end***average* ← *average/n**score* ← *max*{*NormalizeDistance*(*minDistance*) *– α*|*avgGlobal* – *average*|, 0}


The sub-activity algorithm result with the highest score will be the sub-activity selected as recognized for the input sequence. However, all of the scores calculated for all of the activities are transmitted to the high-level activity recognition module composed by the fuzzy ontology, in order to have complete information for complex activity reasoning and inference.

### 3.2. Phase 2: Knowledge-Driven Context-Aware Modeling and Recognition of High-Level ADLs

In this section, we use ontological design principles to represent human activities semantically and uncertainty and vagueness in the representation of information. The semantic inference-based module is based on fuzzy ontological rules [5] and takes as input the sub-activities detected in the first stage and their score, in order to detect high-level activities. Sub-activity scores are considered as a degree of certainty of activity detection, which is used to provide a reliable prediction and ontological reasoning considering uncertainty.

#### 3.2.1. Fuzzy Ontologies for Semantic High-Level Activity Recognition

In many scenarios, and particularly in the human behavior representation domain, we find elements whose nature is imprecise. A classic crisp ontology cannot represent this type of information, since they can only model relations between entities that may be either true or false. Contrary to classical set theory, where elements either belong to a set or not, in the fuzzy set theory [45], elements can belong to a set with some degree. Formally, a fuzzy subset A of X is defined by a membership function *μ*A(x), or simply A(x), which assigns any x & x to a value in the real interval between zero and one. Fuzzy logic allows one to perform approximate reasoning involving inference rules with premises, consequences or both of them containing fuzzy propositions [46].

A fuzzy ontology is an ontology that uses fuzzy logic to provide a natural representation of imprecise and vague knowledge and eases reasoning over it. Fuzzy description logic (DL) is the most developed formalism to work with fuzzy ontologies [30]. Formally, a fuzzy knowledge base (FKB) or fuzzy ontology can be considered as a finite set of axioms that comprises a fuzzy ABox A and a fuzzy TBox T [47]. A fuzzy ABox consists of a finite set of fuzzy (concept or role) assertions, while a fuzzy TBox consists of a finite set of fuzzy general concept inclusions (fuzzy GCIs), with a minimum fuzzy degree of subsumption. Fuzzy ontologies and fuzzy extensions of DL have been shown to be useful in applications from information retrieval and image interpretation to semantic web and others [47].

#### 3.2.2. *fuzzyDL* Reasoner

We consider *fuzzyDL* to be the most convenient existing tool for ontological reasoning with uncertainty. The main features of the *fuzzyDL* reasoner [46] are the extension of the classical description logic *SHIF*(**D**) to the fuzzy case. It allows fuzzy concepts with left-shoulder, right-shoulder, triangular and trapezoidal membership functions, general inclusion axioms and concept modifiers. Fuzzy modifiers may be applied to fuzzy sets to change their membership function. *FuzzyDL* supports crisp intervals that can serve to define fuzzy concrete predicates. In fuzzy rule-based systems (e.g., the Mamdani IF-THEN system), fuzzy IF-THEN rules are fired to a degree, which is a function of the degree of match between their antecedent and the input. The deduction rule is generalized modus ponens. *FuzzyDL*'s reasoning algorithm [46] uses a combination of a tableau algorithm and an MILP (mixed integer linear programming) optimization problem.

In *fuzzyDL*, concept C is satisfiable iff there is an interpretation 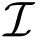 and an individual *x* ∈ Δ; **^
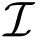
^**, such that *C***^
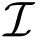
^**(*x*) > 0 [46]. For a set of axioms 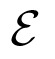, we say that 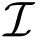 satisfies 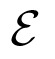 iff 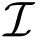 satisfies each element in 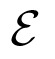. 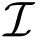 is a model of *E* (resp. 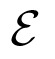) iff 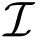 ⊨ *E* (resp. 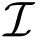 ⊨ 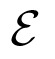). 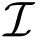 satisfies (is a model of) a fuzzy KB 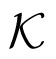 = 〈 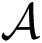, 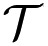, 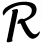 〉, denoted 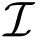 ⊨ 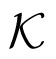, iff 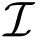 is a model of each component 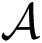, 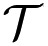 and 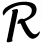, respectively.

An axiom *E* is a logical consequence of a knowledge base *K*, denoted 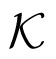 ⊨ *E* iff every model of 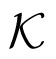 satisfies *E*. Given 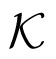 and a fuzzy axiom *τ* of the forms 〈*x* : *C*, α〉, 〈(*x*, *y*) : *R*, *α*〉 or 〈*C* ⊑ *D*, *α*〉, it is of interest to compute *τ*'s best lower degree value bound.

The greatest lower bound of *τ* w.r.t. 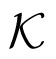 (denoted *glb*( 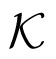, *τ*)) is *glb*( 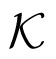, *τ*) = sup{*n*| 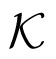 ⊨ 〈*τ* ≥ *n*〉}, where sup ∅ = 0. Determining the *glb*, *i.e.*, the best degree bound (BDB) problem, consists of determining the best satisfiability bound of a concept C:

$$\mathrm{glb}(K,C)=\sup_{I}\sup_{x\in \Delta^{I}}\left\{C^{I}\left(x\right)|I=K\right\}$$

Reasoning tasks allowed by *fuzzyDL* are typical best degree bound (BDB), concept satisfiability and subsumption problems, optimization of variables and defuzzifications.

#### 3.2.3. Ontological Knowledge-Based Algorithm for High-Level Activities

The high-level activity recognition algorithm consists of a series of pre-processing steps before applying ontological reasoning itself. Three basic steps can be distinguished in the knowledge-based algorithm. They cover different aspects from the activity recognition process: First of all, a high-level activity is composed of a sequential execution of a series of sub-activities, where each of these may use some object(s). When recognizing the activities, some of the sub-activities that compose it may be of more relevance within the performance of that activity than others. For instance, some sub-activities may be optional, and some users may not perform some of the most common sub-activities. Therefore, it is needed to learn and compute the weights regarding the importance of each sub-activity in the recognition of each high-level activity. This is done in Step 1.

In the second step, these sub-activity weights will be used in order to create specific rules that represent knowledge in the *fuzzyDL* KB. Finally, a heuristic engine is needed to recognize the activities and deal with issues such as: (1) recognizing activities of different duration (measured in number of sub-activities); (2) sequences of sub-activities patterns that may be contained in the definition of more than one activity; (3) activities that have very characteristic object usage, which makes them easy to discriminate from others. As some activities use almost the same objects, the same subset and/or the same sub-activities, the heuristic engine is crucial to distinguish among activities. Therefore, the engine was designed to discriminate and disclose each activity from the rest, according to a set of ordered filters and considering as much evidence as possible from sub-activities and object interactions. This evidence is used, in some filters, in the form of manually codified sequences of sub-activities that characterize an activity. The heuristic engine is implemented in the form of a pipeline, where each step in the pipeline is a filter that selects those activities that are most probable to be happening, according to the evidence of discrete events. One of the filters in the middle of the pipeline is invoking the *fuzzyDL* inference reasoner to obtain the certainty (or degree of truth) of activities happening. The rest of the filters will help discern the activity that is to be recognized.

Each discrete event is input to the ontological algorithm. At the same time, this input is an output from the data-driven sub-activity recognition module (Algorithm 2). Each atomic event is composed of a pair of a sub-activity and the objects used while it was performed. The detailed steps to recognize high-level activities are described next.

- **Step 1: Learn the weights for each sub-activity within an activity**
  Common sense knowledge is used to define domain knowledge, in our case to define the home activities and the sub-activities that compose each of them. As we perform cross-validation to evaluate our rules on previously unseen users, we compute the value of the sub-activity weights for each sub-activity within an activity, based on the training dataset for certain users, and then test with the new unseen user.
  Sub-activity weights emphasize the importance of each sub-activity within the execution of an activity. Weights are computed based on a naive approach in a previous configuration phase, on a semi-supervised manner, accounting for each sub-activity appearance within the activity performed in the labeled dataset. In other words, sub-activity weights represent the percentage or ratio of how much a sub-activity participates in the execution of a given activity. The average weight is computed and normalized [0,1] considering all sub-activities equally important, e.g., the weight of sub-activity *a_j_* within the activity *A_i_* is computed as in Equation (3):
  (3) $$\frac{\#a_{j}}{\sum_{i=1}^{n}\#a_{i}}$$
  where #*a_j_* is the number of occurrences of sub-activity *a_j_* within activity *A_i_*, and *n* represents the amount of different sub-activities that participate in the given (high-level) activity *A_i_*. Object interactions are associated with each sub-activity, and similarly, all objects initially have the same importance within the activity. Therefore, in the activity rule definition, all possible objects associated with a sub-activity within a given activity are OR-ed. Examples of rules with different objects can be seen in Tables 2 and 3. For instance, this OR-relation is used when defining the sub-activity *reachMicroOrCloth*. The following *fuzzyDL* expression indicates that the pair composed by the sub-activity *reaching* and the used objects *microwave* or *cloth*, is defined by any user which performs the sub-activity *reaching*, while using, at the same time, either a *microwave* or a *cloth*:*(define-concept reachMicroOrCloth (g-and User (some performsSubActivity (g-and reaching (some usesObject (or microwave cloth))))))*The usage of the OR relation in the definition of a sub-activity-object pair, such as in the definition of *reachMicroOrCloth*, is used if there is no unifying object category for all potential objects allowed to be used in that specific sub-activity Let us provide an example where such an object category, which hierarchically unifies the use of several kinds of objects, is used. For instance, the sub-activity-object pair *moveDrinkingKitchenware*, is defined as any user that performs the sub-activity *moving*, while using any kind of object that inherits from the class *drinkingKitchenware*:*(define-concept moveDrinkingKitchenware (g-and User (some performsSubActivity (g-and moving (some usesObject drinkingKitchenware)))))*Tables 3 and 4 show how object categories were defined to group possible types of objects to be used within an adaptable activity definition, in a semantically logical way. Object categories allow one to consider different object usage, e.g., variations from person to person, or just different object usage associated with a given sub-activity. Categories were defined by observing the dataset, using commonsense knowledge, and creating a class for each category in the ontology that inherits from the class *object*. For instance, we define in the fuzzy ontology the *drinkingKitchenware* class as a type (subclass) of *object*. Defined objects, inheriting from this class, are *bowl* and *plate*. Thus, all objects that represent *drinkingKitchenware* are defined as inheriting classes of this class. Let us see in the *fuzzyDL* syntax how this is defined:*(define-concept bowl (and kitchenware stackable movable drinkingKitchenware containerKitchenware)) (define-concept cup (and kitchenware movable drinkingKitchenware containerKitchenware)).*
  The lines above define the object *bowl* as an object of type *kitchenware*, *stackable*, *movable drinkingKitchenware* and *containerKitchenware*. Analogically, the *cup* object is defined with multiple inheritance. In this case, the object category *drinkingKitchenware* is defined semantically as a type of *object*:*(define-primitive-concept drinkingKitchenware Object).*
  If new objects are integrated in the environment, for the system to keep working, only a new definition (as above, for *bowl*) would be needed. This would make explicit the object categories to which the object belongs. Likewise, an associated weight for the sub-activity-object(s) pair would need to be added to the ontology rules, for the activities affected.
- **Step 2: Create rules and represent fuzzy knowledge in the knowledge base**
  Activities are characterized by a set of previously configured sub-activities, in certain sequential order, that are required for the activity to be recognized. Each activity is recognized through a rule that contains a set of axioms involving sub-activities and the objects that these sub-activities use (these rules would be defined by an expert in classical expert systems; in our case, we use natural language and common sense descriptions for each activity, as well as observations from the dataset). Because OWL (Web Ontology Language) does not allow order comparisons among two data properties' literal values in order to consider order among the sub-activities' timestamps, the heuristic engine filters take care of these sequential patterns, prior to executing the reasoner inference engine.
  In this phase, we use the learned weights from Step 1 and each common sense rule to represent an activity. Rules are defined using ontological knowledge representation, which is fed to the KB in the *fuzzyDL* reasoner. The syntax of rules and the formal specification of concepts and relations for each activity, sub-activity, user and object in *fuzzyDL* can be seen in Table 5.
- **Step 3: Run the heuristic engine to recognize an activity**
  Once the KB and rules are defined, ontological inference can take place each time there is a change in the KB, e.g., a new sub-activity occurs or every certain time interval. Semantic web-based reasoning is known to be powerful, but computationally expensive. However, due to recent advancements in the state-of-the-art reasoners, description logics have become more accessible and allow queries' responses to happen in seconds [39].
  As we aim at recognizing critical activities online, *i.e.*, in real-time, assuming a potentially high and rich traffic of multimedia events, we pre-filter some activities that are more prone to happen, based on some heuristics, such as the probability of the activity to happen (based on the labeled occurrences of sub-activities). This is an example of a first heuristic, which together with the following ones, optimizes reasoning and recognition tasks using rich sensor streams. We will next describe each heuristic filter and how it selects or discards activities prone to be detected. If a filter does not select any candidate activity, the candidate activities, from the previous filter applied, are selected. This allows for propagation of the heuristics applied, so that the next filter can be executed, taking as input the previous filter's output candidate activities.
  The **pre-filter ratio**computes the proportion of sub-activities occurring in the most recent time window, from those required by the activity being evaluated. With “most recent time window”, we refer to the most recent time window occurring in the discrete event stream-based dataset. This means that we query for all possible activities that could have happened during the most recently occurring timed events (the last set of sub-activities with start and end timestamps). The pre-filter selects those activities whose pre-ratio value is over a minimum threshold value. This threshold in [0, 1] is computed manually in an empirical form.
  This heuristic considers the activities with the highest proportion of unordered pairs of sub-activities and their associated object interactions. For instance, for the activity *making cereal*, the set of sub-activities (sub-activity-object(s) pairs) that compose it are:*moveMilkOrBowlOrBox, placeMilkOrBowlOrBox, reachMilkOrBowlOrBox, openMilkOrBox, reachMilkOrBowlOrBox, moveMilkOrBowlOrBox, pourMilkOrBox, moveMilkOrBowlOrBox, placeMilkOrBowlOrBox, reachMilkOrBowlOrBox, moveMilkOrBowlOrBox, pourMilkOrBox, moveMilkOrBowlOrBox, placeMilkOrBowlOrBox.*
  Activities filtered in pre-filter ratio are fed, in pipeline, to Filter 1. The Filter 1 heuristic considers, similarly to the pre-filter, the activities with the highest proportion of ordered pairs of sub-activities and their associated object interactions. In this case, Filter 1 assumes an ideal given order.
  Filter 1's output activities (in its default, the pre-filter ratio's output activities) are applied to Filter 2, which considers manually pre-defined order restrictions among (possibly repetitions of) subsets of pairs of sub-activities and objects used and then keeps as candidates those activities with the highest ratio of these subsets. The specification of the order restrictions among subsets of pairs containing sub-activities and objects was done *ad hoc*, using common sense, but mainly by observing the dataset to perceive the sub-activities realized, as well as the objects used in each activity. Since the CAD-120 dataset authors did not provide a more detailed specification in natural language for each of the activity instructions given to the users that were recorded performing each activity, a “manual” observation of the dataset's employed objects was required. We can note that in the original paper [27], only a description for one activity was given, and the rest was assumed to be self-explanatory by the high-level activity name.
  In order to represent semantically order constraints, the optionality of subsets of sub-activities-objects and the number of repetitions of these, we define the concept, activity subsequence. Each activity subsequence is defined through the following triple: *(list of sub-activity-object pairs, optional, minNOfReps)* . The first element indicates a list of sub-activities-object pairs. The second element is a property that indicates if the whole activity subsequence is optional to recognize that activity pattern, and the third element represents the minimum amount required of repetitions of that activity subsequence, to be found in the activity stream. For instance, the pre-defined order restrictions among activity subsequences, for the activity *eating meal*, are defined as follows:*(reachCup, true, 1) (moveCup, eatCup, moveCup, false, 2) (nullSA, true, 1) (reachCup, true, 1) (moveCup, drinkCup, moveCup, false, 2) (placeCup, true, 1)*
  After the heuristic pre-selection filters are applied, the amount of queries to the reasoner is reduced. *FuzzyDL* is queried for the certainty of a set of activities happening, *i.e.*, only those candidate activities that are the output of the pre-filter, Filters 1 and 2. Querying *fuzzyDL* is named in the algorithm as Filter 3.
  Next, the Filter 4 heuristic takes the highest certainty activity considering subsumption properties and concrete restrictions among the used objects' relative positions. The object position filter is only used in the case that there is more than one candidate activity characterized by the same set of sub-activities and objects. For instance, *stacking* and *unstacking objects* are examples of such condition. The activity *stacking* is detected after finding at least three objects of the same type in the unstacked position and, after that, the same type of objects in the stacked position. The activity *unstacking* objects is detected when the same conditions, in opposite order, occur.
  The dataset is very challenging, since different objects can be used within the same activity (e.g., bowls, plates or pizza boxes for eating meal) and the same objects can be used for different activities (e.g., a microwave is used for takeout food or cleaning). Stacking and unstacking activities were performed with pizza boxes, plates and bowls. Furthermore, the order of actions are not always the same (sometimes, one takes the water glass before the pill when taking medicine or the other way around), and some sub-activities are optional in some cases (e.g., in making cereal, milk and cereal boxes do not always need to be opened, but only when there is a new package). Furthermore, when eating a meal, not always does the person drink something at the same time. There may also be other objects the user interacts with in between, and thus, it is not easy to model non-deterministic behavior. Furthermore, e.g., in the activity eating meal, the sub-activity of drinking and eating may be repeated an undefined number of times, for each user and time of day. There are right and left-handed users, and in general, activities are not always performed in the same way. As some objects participate in different activities, it often happens that several activities have the same or very similar certainty of happening. In that case, we select, if any, the one that is a super-activity or that subsumes the other, regarding the sub-activity-object pairs that the activity definition contains. If no activity subsumes another, then the activity that subsumes the other regarding the longest duration, in number of sub-activities, is finally recognized. In order to disclose if there is an activity *A_i_* that subsumes another *A_j_*, we define the binary relation *ContainedIn*(*A_i_*, *A_j_*) as a property that holds when all sub-activities in activity *A_i_* appear within *A_j_*'s activity definition; e.g., in the CAD-120 dataset, this property holds for *ContainedIn(takeout, microwaving)* and *ContainedIn(bending, arrangingObjects)*. This property helps in discriminating very similar activities that otherwise would often be recognized wrongly, e.g., *microwaving* and *arranging objects*, which are usually recognized as *takeout food*.
  Summarizing, if there is a draw, or the activities with the two largest certainty values differ in a small difference , the following heuristic within Filter 4 is applied:
  (1) Select the activity that subsumes other activity in its specification, by sub-activity (SA), *i.e.*, by having exactly the same SAs and object pairs. If not found, (2) is applied.
  (2) Select the activity that subsumes others by cardinal of SAs (the activity's length). Assuming no concurrent activity happens and no activity subsumes another (Case (1)), check if, from the candidate activities, there is one which has the specification of a longer cardinal of SAs than the rest. This favors the recognition of more complex activities if several activities of diverse length are final candidates. If no activity is found, (3) is applied.
  (3) Select the activity with the highest overall ratio, which is computed as an equally ponderated ratio of previously applied filter ratios (pre-ratio, Filter 1 and Filter 2).

The AR algorithm described in Algorithm 3 summarizes all heuristic filters described. Each input data takes the following values:[*Sub-activity, sub-activity ID, video start and end frames, current frame, object ID, object name, left and right hand distances to the object and objectPosition (X, Y, Z axis)*].

**Algorithm 3:** Semantic high-level activity recognition
**Input:** A sequence of sub-activities (associated with objects used) and their detection certainty (from Algorithm 2), in the form of a data stream**Data:** A knowledge base *KB*is given with a set of users, activities, sub-activities and object definitions. A set of rules *R* = {*R*_1_, *R*_2_, … ., *R_n_*}, one per activity, is codified. Rules contain average weights for each associated sub-activity and object pair.A time window *TW*is provided. Its duration size is proportional to each activity that is being considered as a candidate to be recognized. *ActivityRecognitionThreshold*: mininum activity recognition certainty threshold to recognize an activity, given fuzzyDL reasoner's answer.**Result:**
*detectedActivity*: the recognized activity for the input TW (consisting of sequences of sub-activity-object pairs).PRE-FILTER RATIO: Unordered sequence of sub-activity-object pairs *detectedActivities* ← *filter Activities Containing SubActivity Object Pair Constraint s*(*TW*)RATIO 1 FILTER: Ordered sequences of sub-activity-object pairs *detectedActivities*1 ← *filter ActivitiesFul filling Order And Object Constraints* (*detectedActivities*)RATIO 2 FILTER: Activity sub-sequences (of sub-activity-object pairs) *detectedActivities*2 ← *filter Activities Fulfilling Order And Object SubSequence Constraints (detectedActivities1)*RATIO 3 FILTER: Find degree of certainty (*fuzzyDL* min-satisfiability degree query) for each candidate activity**for**
*ActivityAi in detectedActivities2*
**do** *addgetCertaintyOfActivHappening* (*A_i_*)*toactivitiesCertainties* **if**
*getCertaintyOfActivityHappening*(*A_i_*) >*ActivityRecognitionThreshold*
**then**  add *A_i_* to *candidateActivities* **end****end****if**
*candidateActivities.size*() >0 **then** FILTER 4: Find relative object position-based activities, subsumed activities (and overall rate filter in case of certainty, draw among several activities) *detectedActivity* ← *getActivityWithHighestCertaintyOverThresholdactivitiesCertainties, candidateActivities, Activity RecognitionThreshold*)**end****return**
*detectedActivity*


## 4. Semantic Treatment of Uncertainty, Vagueness and Incompleteness in Context-Aware Activity Recognition

There are potentially as many sources of uncertainty as there are sources of information. Furthermore, human natural language, which is subjective and imprecise, also allows for vague expressions when modeling common sense rules or knowledge. As human nature is non-deterministic and humans perform complex behaviors, uncertainty, incompleteness and vagueness are unavoidable aspects to consider when modeling human activities. These are inherent components of human behavior that need to be accounted for. Next, we describe what the source of uncertainty can be in AmI environments and how they can be treated in our approach.

### 4.1. Incompleteness in Missing Sensor Readings

Missing or failing sensor readings is one of the most typical sources of uncertainty in AR [48]. A concrete ambient assisted living (AAL) experiment in [48] showed that 51% of the system crash reasons were due to sensors being out of battery, 22% due to packet lost, 12% due to a reasoning failure, 8% due to sensors being removed and 7% because of WiFi disconnection. By using a fuzzy ontology, a missing sensor reading does not drastically affect the recognition of a pattern, but rather diminishes the certainty of satisfiability of a given activity to be recognized as happening. This is thanks to axiom definitions based on weighted concepts, which express the importance of a sub-activity associated with an object interaction. Fuzzy logic allows for flexibility or looseness in the model by accounting for the rest of the components, if one in the definition is not instantiated.

### 4.2. Uncertainty in Sensor Data Acquisition

Pipeline-based or multi-level activity recognition has the disadvantage of incurring error propagation. However, fuzzy ontologies allow declaration of axioms with a given truth degree for each atomic element (e.g., each sub-activity detected or each recognized object). Given the 3D-depth sensor certainty to recognize a sub-activity, a sub-activity instance can be input into the knowledge base to indicate its recognition with a certainty degree in [0, 1]. For instance, in *fuzzyDL*, stating that a sub-activity instance of type *placing* is detected with a degree of truth of 0.5 is defined as follows: e.g., *(instance placing subActivity 0.5)*.

### 4.3. Vagueness in the Importance of Each Sub-Activity within a High-Level Activity

Not every user performs an activity in the same way. Some users change the predefined order in which they perform each sub-activity, and other users may skip some sub-activities depending on the context or use different objects depending on preferences or situations (e.g., while eating, there is not a fixed predetermined number of repetitions for the sequence related to bringing the cutlery close to the mouth). These uncertainty aspects leave us room for abstraction when representing knowledge. We base our model or activity pattern on common sense knowledge and observations from the dataset. Even when modeling these uncertain criteria, the semantic model should, in any case, maximize the degree of satisfiability or similarity to the defined fuzzy concept definition of activity. As indicated earlier, weights associated with the importance of each sub-activity within an activity definition, for each cross-validation fold in our experiment, were taken from the dataset. However, if no evidence would exist, it is possible for the domain expert to set them *ad hoc*.

### 4.4. Other Vagueness and Uncertainty Sources in Activity Recognition

Identifying the right user performing an activity is crucial to detect critical activities, as well as distinguishing among possible activities being performed concurrently. In multi-user scenarios, 3D-depth sensors are expected to achieve very significant improvements in the very near future and to reduce noise, e.g., in face or body recognition. These are other kind of uncertainty to be dealt with in the data acquisition phase. In our fuzzy ontology, we can state the certainty degree with which a user is identified, e.g., in *fuzzyDL*, *(instance Natalia User 0.9)* means that Natalia is an instance of the class Userwith a degree of truth of 0.9. We can also express the certainty with which the system identifies or recognizes a concrete user performing an activity. For instance, in *fuzzyDL*, *(related Natalia traveling performsActivity 0.9)* means that Natalia performs the activity traveling with a certainty degree of 0.9. These are just two examples of how any possible axioms can be upgraded by including an uncertainty degree *dimension*.

Detecting object interaction is another key context-aware component to discriminate among activities. However, the proximity of the user to objects does not always imply interaction. The closeness of the user's hands to the objects, as well as the relative distance among objects are key to distinguishing among activities that use the same (sub)sequences of sub-activities and the same kind of objects (e.g., in CAD-120, *stacking and unstacking objects*). Therefore, *DistanceToHands and maxDistanceAlongYAxis* are samples of thresholds used programmatically to deal with measurement and error variations. Likewise, the time window needs to adapt its size to a threshold-based buffer when querying for certain activities. In our case, we used a threshold summed to the maximum execution time of a given activity. However, a fuzzy temporal window to express the times of the day when an activity can happen can also be utilized [49].

## 5. Experiment and Validation of the Hybrid AR Framework

### 5.1. CAD-120 3D-Depth Dataset

Although the literature offers a wide variety of activity recognition datasets, it is hard to find one with a enough diversity to test fine- and coarse-grained activities in RGB-D video and where semantics features can be tested, together with object interaction, to allow discrimination of activities according to context. The dataset that best suits our requirements for different levels of activity recognition is the recent CAD-120 dataset (Cornell Activity Dataset) [27]. It is a very challenging dataset that contains 120 activities with 1,191 sub-activities performed by four subjects: two male and two female (one of them left-handed). It contains the following high-level activities, sub-activities and labeled objects:

- Ten high-level activities: making cereal, taking medicine, stacking objects, unstacking objects, microwaving food, picking objects, cleaning objects, taking food, arranging objects, having a meal.
- Ten sub-activity labels: reaching, moving, pouring, eating, drinking, opening, placing, closing, scrubbing, null.
- Ten objects: book, bowl, box, cloth, cup, medicine box, microwave, milk, plate, remote.
  The goal of our experiment is to test the performance of our approach under complex AR scenarios by adding semantics, through fuzzy ontology-based context reasoning, to data-driven AR. With that purpose, we define the parameters in Equations (4)–(6), where *tp* stands for true positives, *tn* for true negatives, *fp* for false positives and *fn* for false negatives.
  (4) precision=tp/(tp+fp)
  (5) recall=tp/(tp+fn)
  (6) accuracy=(tp+tn)/(tp+tn+fp+fn)
  As a second evaluation metric, we are interested in the scalability of the system, as a reactive system. Scalability is understood as the capability of the ontology to perform with a rule set and a reasoner to achieve AR, in reasonable execution time, for large amounts of data (KB' size). The system was shown to be scalable in this sense [5], but in this case, we aim at having a whole hybrid AR system that can assist users, responding to changes or special situations in the environment, in real time. For this reason, we use a metric that is critical for system responsiveness in order to assess the real-time system performance for continuous AR [36]: the time per recognition operation (TpRO) is defined as the interval in seconds from the time a sensor is activated until the time an activity is recognized. The results of our approach, for these metrics, in each of the two phases of the algorithm, are shown in next subsections.
  We performed leave-one-out cross-validation in each of the two phases as in *Koppula et al*. [27] (CAD-120 dataset's authors), using each subset of three users for training and tested with the fourth one, to be able to compare our approach with the previous method accurately.

### 5.2. Evaluation of the Data-Driven Recognition of Sub-Activities

Table 6 shows the average results of the cross-validation process for sub-activity labeling.

We have also measured the recognition execution time for each sample and calculated the average. These experiments were done running the developed application over Ubuntu 12.10 as the OS, on a PC with a Pentium Dual-Core processor (CPU E5700, 3.00 GHz, 800 MHz FSB , 2 GB RAM). Each sub-activity sample has a different duration, varying from 10 to 510 frames. There were 11 sub-activity samples, out of 1191, in the CAD-120 dataset with a duration lower than 10 frames (one third of a second), but we have discarded these ones, as we think they are possibly due to some misprints when labeling the data, as their length greatly differs with the average of their kind. We present these results on Table 7. As can be observed, the average sub-activity recognition time is 178.99 milliseconds, and since the average sub-activity duration is 50.8 frames, this means our recognition algorithm is able to process more than 380 frames in less than one second, in a medium range of a five-year-old CPU.

Table 8 shows the results obtained for the experiment carried out. We consider the comparison with the basic method, where Koppula *et al.* [27] obtained 76.8% average accuracy, 72.9% precision and 70.5% recall (overall on average with ground truth temporal segmentation and object tracking). We observe an increment in the results with the solution we propose. This is due to the usage of our framework described in Section 3.1, which achieves 90.1% average accuracy according to the results shown previously. Thus, the approach presented in this work is highly competitive.

In order to verify these results, we applied a statistical analysis to evaluate if the improvement is statistically significant. The null hypothesis of equal performance between classifiers is rejected according to the Student's sample *t*-test for *α* = 0.05 with a *p*-value of 7.3632e-04. As the hypothesis has a *p*-value ≤0.05, there is a statistically significant difference in improvement between Koppula *et al.* [27] and our recognition algorithms.

### 5.3. Evaluation of the Knowledge-Based Recognition of High-Level Activities

The main features of the CAD-120 dataset were adapted to ontological concepts and relations. In order to semantically define an activity, different concepts and relations must be explicitly defined, as well as the order of the relations among sub-activities. For this purpose, we use the given high-level descriptions of the activities that users were asked to perform (multiple times with different objects). For example, the instructions for making cereal were: (1) place bowl on table; (2) pour cereal; (3) pour milk. A summary of the rest of activities is described in Table 9.

Object interaction in the ontology was modeled by categorizing each object by its usage in order to discriminate among activities. These semantic categories or super classes are in Table 10, while the object and data properties modeled are in Table 11.

When it comes to populating the ontology with real data, instances of each class, called individuals, are created. Individuals are created in OWL through an instantiation in the form of an RDFtriple (subject, predicate, object). Ontological relations represent a property among two classes (called object properties) or among a class and a data-type (called data properties). Therefore, the content in Table 11 specifies the ontological schema in which instances (in the form of triples) can be represented, conforming to the types specified by the subject (the domain of the relation), the predicate (the object/data property) and the object (the data-type range of the relation). Once provided, these values, in a way that the ontology remains consistent, the reasoning engine can perform an inference of new relations and properties automatically.

The rules created for the ontology validation experiment are shown in Table 5, and the concepts, properties and axioms that the rules use are defined in Tables 2 and 4 and 11. Additional information about the *fuzzyDL* syntax may be found on the fuzzyDL website (*fuzzyDL*: http://gaia.isti.cnr.it/straccia/software/fuzzyDL/fuzzyDL.html).

We believe that this dataset is accurate and close to real scenarios, because objects are used in different tasks with different purposes, and the same activity could be performed with different objects. For example, the stacking and unstacking activities were performed with pizza boxes, plates and bowls. Activities were performed through a long sequence of sub-activities, which varied from subject to subject significantly in terms of the length of the sub-activities. The order in which the sub-activities were executed within a task can also differ.

Confusion matrices in Tables 12 and 13 show in detail the accuracy values for each activity with two settings: firstly, in combination with the data-driven module that returns the detected activity and, secondly, replacing the data-driven module with the ground-truth sub-activity detection. These two settings help to verify the influence on the accuracy with respect to the low-level activity recognized and also to know the limits of our fuzzy ontology approach.

Regarding the performed experiment, Table 14 shows the results obtained. We set as the baseline the method proposed by Koppula *et al.* [27], using the CAD-120 dataset, which results in a 79% accuracy, 78.6% precision and 78.3% recall overall on average. We appreciate an improvement in the knowledge-based solution that we propose, increasing the average accuracy to 82.9% and the precision and recall, respectively, to 84.1% and 97.4%.

In order to evaluate the effects (or costs) of the manual common-sense knowledge codification work, reflected in the usage of the heuristic in Algorithm 3, we performed a naive implementation, where no heuristic filters were applied. Instead, we solely applied the *fuzzyDL* reasoner to find out the activity with the highest certainty of being predicted (in this setting, only the pre-filter ratio in Algorithm 3 was used, which provides information to query only for those activities for which any kind of evidence has occurred in the last event time window, *i.e.*, we only query for activities for which any of their involved sub-activities has occurred). Results are also shown in Table 14, and the respective confusion matrix is in Table 15.

Avoiding the application of the heuristic filters provides poor results when compared with the whole hybrid system, and therefore, their absence makes the method incomplete. In this case, too high a rate of false positives and negatives was obtained for the recognition of high-level activities. This is explained by the fact that DL reasoners are based on monotonic logic, which means that the addition of new axioms into the KB can be performed, but no retraction of information takes effect. In other words, the certainty of an axiom can be increased, but not decreased. The inability to retract facts within a time window of a given size requires the manual pre-definition of the sub-activity “profile” filters. These filters required common-sense knowledge and observation of the dataset to determine a set of subsequences of sub-activities and object patterns in each activity. In the heuristic filters algorithm, after querying the *fuzzyDL* reasoner for each activity's satisfiability degree, the filters are applied to avoid too high of a false positive rate and a false negative rate, as well as for improving the accuracy, precision and recall metric values. Too high a false positive rate would be even more noticeable in potential cases, such as multi-users, parallel or interleaved AR settings (which is not the case for CAD-120). In any case, logic monotonicity is inconvenient to deal with when using DLs, and future solutions should be sought for larger stream data flows.

On the other hand, if we assume an ideal scenario with 100% accuracy on labeled input sub-activities, *i.e.*, supposing all sub-activities are properly recognized in the first phase, then a precision of 90.8%, a recall of 98.1% and an accuracy of 91.07% are achieved. Both experiments in the sub-activity and high-level activity tracking and recognition (both in CAD-120 and our framework) were realized following the settings in Koppula *et al.* [27], *i.e.*, assuming ground truth temporal segmentation is given.

Furthermore, we executed a statistical analysis in order to evaluate if our improvement is statistically significant. The null hypothesis of equal performance between classifiers is rejected according to the sample Student's *t*-test for *α* = 0.05 with a *p*-value of 0.0013. As the hypothesis has a *p*-value ≤ 0.05, there is a statistically significant difference between Koppula *et al.* [27] and our recognition algorithms. In addition, our average standard deviation from all mean precision values is smaller than the one for Koppula *et al.* [27], as we have *σ* = 0.0092, while Koppula *et al.* [27] produce *σ* = 4.1 for the full model with tracking capabilities in the real-life scenario (non-ideal case with predicted sub-activities as the input). Final overall comparison statistics are shown in Table 14.

Regarding AR execution times, we run the experiment on an Intel(R) Core i7-4500 @ 1.80 GHZ 2.40 GHZ, 8 GB RAM 64-bit and Windows 8.1 OS. Table 16 shows that our high-level AR time (TpRO) averages 0.56 seconds. Thus, this timing makes our approach closer to real-time than existing solutions. Reported results in similar settings were found in the literature [36] with an average TpRO of 2.5 s.

## 6. Discussion

From the experiments conducted, it can be seen that activities, such as bending, stacking, microwaving and unstacking, are not as well recognized as others. The propagation of errors from the first sub-activity recognition phase is a reason for this, as well as the noise in the detection of the objects and their positions (especially with respect to each other and when occlusions appear). It is worth noticing that the AR is performed taking as input the output of the sub-activity recognition module that, at the same time, performs real-time (user and object) tracking. This adds an extra challenging dimension to the hybrid system, and therefore, the results presented are still promising. With the upcoming advances of a new generation of RGB-D sensors, these problems are expected to be solved, and therefore, the heuristics used in the high-level AR phase are expected to produce better detection results.

## 7. Conclusions and Future Work

Knowledge-based techniques, such as ontology-based activity modeling, add a set of advantages for incremental and context-aware recognition. It is a suitable approach to achieve interoperability, abstraction and modularity in an easier way. However, some expressiveness limitations in OWL DL are also found related to the lack of support for temporal reasoning [50], and often, external rule engines are used to express more complex modeling. Although ontologies for human activity modeling have been created in the recent past [4] to deal with context information, our proposal models and treats uncertain, incomplete, vague or imprecise information, in a real-life challenging dataset in order to represent more flexible models. Realistic missing sensor readings due to, e.g., occlusions or sensor failures, are tackled in this way naturally.

The advantage of ontologies and, in general, knowledge-based methods is that expert knowledge can be introduced directly in the knowledge base, while data-driven approaches require a great amount of initial data, training of the model and validation. This fact, therefore, requires the inclusion of common sense knowledge in the ontology in order to palliate the need for large amounts of data. This can be seen as an advantage when there is not enough training data.

Regarding scalability when adding new classes to the ontology, it has been proven that the system is responsive and scales for large amounts of triples in the knowledge base [5], and thus, it is not a problem when new activities, sub-activities and objects are require to be added to the ontology. If the system needs to recognize new activities, a new class for each sub-activity and activity to be recognized needs to be added to the ontology definition. This is the alternative that knowledge-based methods offer against data-driven ones. It is understood that the former are not totally “training-free” methodologies and that they come with a price to pay, which we believe is small once the data-driven training phase has been achieved within our hybrid system. In order to upgrade knowledge-driven AR systems, such as ours, the updating of sub-activity weights and rule definitions in the ontology is needed. We believe that this is an affordable price to pay in contrast to recording new datasets with diverse users, objects, old and new activities, plus retraining and validating the system.

In summary, our framework provides the following important contributions:

- Tracking and recognition of complex sub-activities and high-level activities involving object interaction from 3D-depth images in a novel, real-life and non-synthetic dataset. Although there exist many crisp ontologies for activity modeling, such as mIO! [51], PalSPOT [50], CONON [52], PiVOn [53] or Situation Ontology [54], there does not exist a fuzzy ontology previous to ours [5], dedicated to activity recognition. Therefore, apart from validating the advantages of our fuzzy ontology, at a theoretical level, previously in [5], in this work, we took a step further. We developed a hybrid system applied at a practical level and used a more complex real life dataset.
- Statistically significant improved results on precision, recall and accuracy of 91.5%, 97% and 90.1%, respectively, for the first data-driven stage of the AR system (sub-activities). We achieved 84.1% in precision, 97.4% in recall and 82.9% in accuracy for the knowledge-driven, ontological and last stage of the AR system, *i.e.*, for high-level activities. Assuming an ideal scenario with 100% correctly labeled input sub-activities, a precision of 90.8%, a recall of 98.1% and an accuracy of 91.07% was achieved.
- Automatic treatment of uncertain, incomplete and vague information with a fuzzy ontology that makes the system (sensor and prediction) fault tolerant. While a crisp system considers axioms true or false in its totality, in a fuzzy system, conditions and facts can occur to a certain degree in [0,1]. This means that we can have degrees of truth for each isolated event, sensor reading, interpretation and build on top of that, considering the uncertainty of each data source independently, but in relation to the satisfaction of high-level concepts. This is handled in our hybrid system thanks to the higher abstraction inference layer through the *fuzzyDL* reasoner. For instance, when any of the sensors used for data acquisition fails or breaks, the activity will not be recognized at all in purely crisp approaches. In contrast to traditionally crisp approaches, uncertainty reasoning provides a lower degree of recognition in [0,1] that still gives a degree of truth for the activity performed, even if a sensor fails, breaks or its reading is not captured. Faults can also occur in data interpretation (e.g., recognizing objects or users), and thus, this is another kind of tolerated fault.
- Reactiveness and scalability: real-time tracking for on-line recognition and deployment in, e.g., AmI or AAL environments.

Our proposal, thus, provides a versatile system with varied (from fine- to coarse-grained) levels of abstraction to detect atomic and complex activities, considering the user's interaction with objects and real-time tracking, as well as uncertain or imprecise data, such as missing sensor observations. The method, based on an ontological rule-based system for the highest abstraction layer of activities, is interpretable and allows new integration or adaptation of new sensors without requiring re-training, but rather just editing the activity modeling rules or the sub-activity weights. As a general contribution, we propose an overall data- and knowledge-driven two-phased algorithm that tackles the challenges of complex AR systems by using the most suitable method in each phase, real-time speed and accuracy for the first stage and real-time provision of contextual meaning for activities in the second phase. The evaluation is done using an external non-synthetic dataset with unlabeled continuous 3D-depth image data.

The purpose of the filters applied in the knowledge-based phase are indispensable for achieving good accuracy and precision results, since their main aim is helping to discriminate among similar activities that use similar objects or activities that use almost the same subsequences of sub-activities in time. These filters form part of the hybrid activity recognition method based on knowledge engineering. This manual specification is a typical characterization required *a priori* in knowledge engineering methods in general, where experts are required to set domain rules for an expert system to produce successful predictions.

Therefore, we can identify some disadvantages in our method. It is not a fully automatic method, since it requires one to include expert knowledge in the KB. However, in some applications, this may not be a limitation, since it is possible to define *ad hoc* activities and, in this way, to reduce the training time needed and the adaptation of the system to different environments. In terms of software design, an inconvenience of the proposal is its complexity compared to methods that only use either knowledge-based or data-driven approaches, for learning and recognizing activities. However, this complexity increase is compensated for by an improvement in the activity recognition rate, as we have seen in the experimental section. Finally, the use of a fuzzy ontology to model knowledge, as opposed to traditional crisp ontologies, can better model the problem and the uncertainty in activities; however, the higher order of complexity of fuzzy reasoners can be a limitation compared to crisp reasoners, when the number of activities in the ontology grow substantially within the knowledge base, as it can require a much greater processing time for the recognition. In [5], we theoretically analyzed this problem for cases up to KB sizes of 10^5^ triples, where crisp reasoning was compared with fuzzy reasoning, and found that for the current application, it would be feasible to use these techniques, obtaining reduced run times, whenever the system is applied to a limited space, such as a home setting. In fact, despite this limitation, in this work, we found that in these cases, it is possible for the system run time to become close to real time.

In the future, our system could be further generalized to recognize multi-user concurrent/interleaved activities, which were not included in the CAD-120 dataset.

Future work should also consider the use of equally expressive fuzzy reasoners, such as *fuzzyDL*, with the added value of supporting the retractability of axioms [48]. This means the ability to allow the deletion of data from the KB in order to update information, since AAL makes use of rapidly changing data sources. This addition would permit the reduction of the computational complexity required in the current approach, in order to empty the database every time a query needs to be done in a different time window. As in description logics a given axiom's certainty value can only be raised and not lowered, with the addition of new axioms, due to the monotonicity property of this type of first order logic, it is unavoidable to empty and reload the KB every time a new time window is considered. This is required to preserve more coherent results and favor the last events that occurred (despite the sliding window's flexible size). A compromise between expressibility, efficiency for large amounts of events and precision must be sought within reasoners. Only in this way will the power, and also the functionality, of automatic uncertainty reasoning be preserved and fully taken advantage of.

## Figures and Tables

**Table 1.** Summary of the features vector used for the dynamic time warping (DTW) algorithm.

**Description**	**Count**
**Skeleton Features**	**4**

- Left and right arm. Joint angle shoulder (*joints elbow-shoulderand shoulder-hip*)	2
- Left and right arm. Joint angle elbow (*joints shoulder-hand elbow-shoulder*)	2

**Objects Features**	**16**

- Shortest distance to hand, group by object type (10 objects type)	10
- Shortest distance to hand, group by object id (maximum number of same objects type: 5)	5
- Sum of objects distances	1

**Table 2.** Excerpt of fuzzy concepts used in the rules.

**Sub-activities definitions**	*(define-concept openMilkOrBox (g-and User (some performsSubActivity (g-and opening (some usesObject (or milk box))))))*
	*(define-concept reachMilkOrBowlOrBox (g-and User (some performsSubActivity (g-and reaching (some usesObject (or milk bowl box))))))*
	*(define-concept moveMilkOrBowlOrBox (g-and User (some performsSubActivity (g-and moving (some usesObject (or box milk bowl))))))*
	*(define-concept placeMilkOrBowlOrBox (g-and User (some performsSubActivity (g-and placing (some usesObject (or box milk bowl))))))*
	*(define-concept pourMilkOrBox (g-and User (some performsSubActivity (g-and pouring (some usesObject (or milk box))))))*
	*(define-concept reachCup (g-and User (some performsSubActivity (g-and reaching (some usesObject cup)))))*
	*(define-concept reachMedicineBox (g-and User (some performsSubActivity (g-and reaching (some usesObject medicineBox)))))*
	*(define-concept openMedicineBox (g-and User (some performsSubActivity (g-and opening (some usesObject medicineBox)))))*
	*(define-concept moveMedicineBox (g-and User (some performsSubActivity (g-and moving (some usesObject medicineBox)))))*
	*(define-concept moveCup (g-and User (some performsSubActivity (g-and moving (some usesObject cup)))))*
	*(define-concept eatMedicineBox (g-and User (some performsSubActivity (g-and eating (some usesObject medicineBox)))))*
	*(define-concept placeCupOrMedicineBox (g-and User (some performsSubActivity (g-and placing (some usesObject (or cup medicineBox))))))*
	*(define-concept drinkCup (g-and User (some performsSubActivity (g-and drinking (some usesObject cup)))))*
	*(define-concept reachCupOrMedicineBox (g-and User (some performsSubActivity (g-and reaching (some usesObject (or cup medicineBox))))))*
	*(define-concept moveCupOrMedicineBox (g-and User (some performsSubActivity (g-and moving (some usesObject (or cup medicineBox))))))*
	*(define-concept reachStackable (g-and User (some performsSubActivity (g-and reaching (some usesObject stackable)))))*
	*(define-concept moveStackable (g-and User (some performsSubActivity (g-and moving (some usesObject stackable)))))*
	*(define-concept placeStackable (g-and User (some performsSubActivity (g-and placing (some usesObject stackable)))))*
	*(define-concept reachMicro (g-and User (some performsSubActivity (g-and reaching (some usesObject microwave)))))*

**Table 3.** Excerpt of fuzzy concepts used in the rules (Part II).

**Sub-activities definitions**	*(define-concept placeCloth (g-and User (some performsSubActivity (g-and placing (some usesObject cloth)))))*
	*(define-concept reachDrinkingKitchenware (g-and User (some performsSubActivity (g-and reaching (some usesObject drinkingKitchenware)))))*
	*(define-concept moveDrinkingKitchenware (g-and User (some performsSubActivity (g-and moving (some usesObject drinkingKitchenware)))))*
	*(define-concept placeDrinkingKitchenware (g-and User (some performsSubActivity (g-and placing (some usesObject drinkingKitchenware)))))*
	*(define-concept openMicro (g-and User (some performsSubActivity (g-and opening (some usesObject microwave)))))*
	*(define-concept closeMicro (g-and User (some performsSubActivity (g-and closing (some usesObject microwave)))))*
	*(define-concept reachMicroOrDrinkingKitchenware (g-and User (some performsSubActivity (g-and reaching (some usesObject (or microwave drinkingKitchenware))))))*
	*(define-concept reachPickable (g-and User (some performsSubActivity (g-and reaching (some usesObject pickable)))))*
	*(define-concept movePickable (g-and User (some performsSubActivity (g-and moving (some usesObject pickable)))))*
	*(define-concept reachMicroOrCloth (g-and User (some performsSubActivity (g-and reaching (some usesObject (or microwave cloth))))))*
	*(define-concept moveCloth (g-and User (some performsSubActivity (g-and moving (some usesObject cloth)))))*
	*(define-concept cleanMicroOrCloth (g-and User (some performsSubActivity (g-and closing (some usesObject (or microwave cloth))))))*
	*(define-concept cleanMicro (g-and User (some performsSubActivity (g-and cleaning (some usesObject microwave)))))*
	*(define-concept cleanCloth (g-and User (some performsSubActivity (g-and cleaning (some usesObject cloth)))))*
	*(define-concept reachContainerKitchenwareOrMicro (g-and User (some performsSubActivity (g-and reaching (some usesObject (or microwave containerKitchenware))))))*
	*(define-concept moveContainerKitchenware (g-and User (some performsSubActivity (g-and moving (some usesObject containerKitchenware)))))*
	*(define-concept placeContainerKitchenware (g-and User (some performsSubActivity (g-and placing (some usesObject containerKitchenware)))))*

**Table 4.** Fuzzy concept definitions.

Primitive Classes	(define-primitive-concept User *top*)
	(define-primitive-concept Object *top*)
	(define-primitive-concept Activity *top*)
	(define-primitive-concept SubActivity *top*)
Sub-activities	*(define-primitive-concept reaching SubActivity)*
	*(define-primitive-concept moving SubActivity)*
	*(define-primitive-concept pouring SubActivity)*
	*(define-primitive-concept eating SubActivity)*
	*(define-primitive-concept drinking SubActivity)*
	*(define-primitive-concept opening SubActivity)*
	*(define-primitive-concept placing SubActivity)*
	*(define-primitive-concept closing SubActivity)*
	*(define-primitive-concept cleaning SubActivity)*
	*(define-primitive-concept null SubActivity)*
High-level activities	*(define-primitive-concept cereal Activity)*
	*(define-primitive-concept medicine Activity)*
	*(define-primitive-concept stacking Activity)*
	*(define-primitive-concept unstacking Activity)*
	*(define-primitive-concept microwaving Activity)*
	*(define-primitive-concept bending Activity)*
	*(define-primitive-concept cleaningObjects Activity)*
	*(define-primitive-concept takeout Activity)*
	*(define-primitive-concept arrangingObjects Activity)*
	*(define-primitive-concept eatingMeal Activity)*
	*(define-primitive-concept nullA Activity)*
Object categories	*(define-primitive-concept kitchenware Object)*
	*(define-primitive-concept stackable Object)*
	*(define-primitive-concept edible Object)*
	*(define-primitive-concept movable Object)*
	*(define-primitive-concept drinkingKitchenware Object)*
	*(define-primitive-concept pickable Object)*
	*(define-primitive-concept containerKitchenware Object)*
	*(define-primitive-concept arrangeable Object)*

**Table 5.** Fuzzy rules for each activity.

**Rule 1 Definition:**	*(define-concept antecedent1 (w-sum (0.17 reachMilkOrBowlOrBox)(0.41 moveMilkOrBowlOrBox)(0.24 placeMilkOrBowlOrBox)(0.01 openMilkOrBox)(0.16 pourMilkOrBox))) (define-concept consequent1 (g-and User (some performsActivity cereal)))*
**Rule 2 Definition:**	*(define-concept antecedent2 (w-sum (0.29 reachCupOrMedicineBox)(0.3 moveCupOrMedicineBox)(0.1 placeCupOrMedicineBox)(0.1 openMedicineBox)(0.1 eatMedicineBox)(0.1 drinkCup))) (define-concept consequent2 (g-and User (some performsActivity medicine)))*
**Rule 3 Definition:**	*(define-concept antecedent3 (w-sum (0.26 reachStackable)(0.27 moveStackable)(0.27 placeStackable)(0.20 nullSA))) (define-concept consequent3 (g-and User (some performsActivity stacking)))*
**Rule 4 Definition:**	*(define-concept antecedent4 (w-sum (0.26 reachStackable)(0.27 moveStackable)(0.27 placeStackable)(0.20 nullSA))) (define-concept consequent4 (g-and User (some performsActivity unstacking)))*
**Rule 5 Definition:**	*(define-concept antecedent5 (w-sum (0.32 reachMicroOrDrinkingKitchenware)(0.11 moveDrinkingKitchenware)(0.11 placeDrinkingKitchenware)(0.12 openMicro)(0.11 closeMicro)(0.23 nullSA))) (define-concept consequent5 (g-and User (some performsActivity microwaving)))*
**Rule 6 Definition:**	*(define-concept antecedent6 (w-sum (0.26 reachPickable)(0.27 movePickable)(0.47 nullSA))) (define-concept consequent6 (g-and User (some performsActivity bending)))*
**Rule 7 Definition:**	*(define-concept antecedent7 (w-sum (0.27 reachMicroOrCloth)(0.23 moveCloth)(0.1 placeCloth)(0.1 openMicro)(0.1 closeMicro)(0.1 cleanMicroOrCloth)(0.1 nullSA))) (define-concept consequent7 (g-and User (some performsActivity cleaningObjects)))*
**Rule 8 Definition:**	*(define-concept antecedent8 (w-sum (0.38 reachContainerKitchenwareOrMicro)(0.12 moveContainerKitchenware)(0.12 placeContainerKitchenware)(0.13 openMicro)(0.13 closeMicro)(0.12 nullSA))) (define-concept consequent8 (g-and User (some performsActivity takeout)))*
**Rule 9 Definition:**	*(define-concept antecedent9 (w-sum (0.23 reachArrangeable) (0.27 moveArrangeable)(0.25 placeArrangeable)(0.25 nullSA))) (define-concept consequent9 (g-and User (some performsActivity arrangingObjects)))*
**Rule 10 Definition:**	*(define-concept antecedent10 (w-sum (0.08 reachCup)(0.45 moveCup)(0.07 placeCup)(0.13 eatCup)(0.1 drinkCup)(0.17 nullSA))) (define-concept consequent10 (g-and User (some performsActivity eatingMeal)))*

**Table 6.** Confusion matrix for sub-activity labeling.

Reaching	0.94	0.01				0.01	0.02	0.01		0.04
Moving	0.02	0.89	0.02	0.01	0.01		0.04			0.02
Pouring		0.02	0.96				0.02			
Eating	0.02	0.06		0.84	0.03					0.05
Drinking	0.02	0.08			0.81		0.02			0.08
Opening	0.03	0.02				0.91		0.02		0.01
Placing	0.02	0.04	0.01				0.90			0.02
Closing	0.03	0.01				0.03	0.01	0.88	0.01	0.02
Scrubbing		0.02				0.02		0.04	0.92	
null	0.02	0.02			0.01		0.01			0.93
	Reaching	Moving	Pouring	Eating	Drinking	Opening	Placing	Closing	Scrubbing	null

**Table 7.** Average recognition times (in milliseconds) per sub-activity.

**Sub-Activity Recognition Time**	**Average Time**
Reaching	138.87
Moving	193.9
Pouring	279.55
Eating	141.33
Drinking	178.5
Opening	284.87
Placing	142.46
Closing	241.58
Scrubbing	532.99
null	173.02

**Average**	178.99

**Table 8.** Comparison of our approach with Koppula *et al.* [27] for the CAD-120 dataset (Cornell Activity Dataset) sub-activity recognition. Average Accuracy, Precision and Recall.

**Method**	**Accuracy (%)**	**Precision (%)**	**Recall (%)**
Koppula *et al.* [27]	76.8 ± 0.9	72.9 ± 1.2	70.5 ± 3.0
Our Method	90.1 ± 8.2	91.5 ± 4.6	97.0 ± 5.8

**Table 9.** Semantic description of each high-level activity.

**Activity**	**Semantic concept**	**Description**
Making cereal	*cereal*	Take cereal box, bowl and milk (open them) and pour both.
Taking medicine	*medicine*	Take medicine box from cupboard, take glass, eat pill and drink water
Stacking objects	*stacking*	Stack on a pile plates, boxes or bowls
Unstacking objects	*unstacking*	Unstack from a pile plates, boxes or bowls
Microwaving food	*microwaving*	Take food container or kitchenware, place it into microwave and take it out
Picking object	*bending*	Pick up an object from the floor
Cleaning objects	*cleaningObjects*	Clean up objects (microwave with a cloth)
Taking out food	*takeout*	Take food and heat in microwave
Arranging objects	*arrangingObjects*	Arranging on a table, e.g., setting up the table
Having meal	*eatingMeal*	Eating a meal on the table

**Table 10.** Objects' semantic descriptions based on usage-driven object categories.

**Object**	**Semantic description based on object category super-concepts**
*book*	*(and arrangeable movable)*
*bowl*	*(and kitchenware stackable movable drinkingKitchenware containerKitchenware)*
*box*	*(and stackable movable pickable arrangeable)*
*cloth*	*(and kitchenware movable)*
*cup*	*(and kitchenware movable drinkingKitchenware containerKitchenware)*
*medicineBox*	*(and edible movable)*
*microwave*	*(Object)*
*milk*	*(and edible movable)*
*plate*	*(and kitchenware stackable movable containerKitchenware)*
*remote*	*(Object)*

**Table 11.** Fuzzy roles (ontology object and data properties).

**Description**	**Object Properties**	**Domain**	**Range**	**Property**
High-level activity performed	*performsActivity*	User	Activity	
Sub-activity performed	*performsSubActivity*	User	SubActivity	
Object interaction within a sub-activity	*usesObject*	SubActivity	Object	

**Description**	**Data Properties**	**Domain**	**Range**	**Property**

Sub-activity start frame	*hasStartFrame*	SubActivity	integer	functional
Sub-activity end frame	*hasEndFrame*	SubActivity	integer	functional
Object position in X-axis	*hasPosX*	Object	*double*	0
Object position in Y-axis	*hasPosY*	Object	100,000 *double*	0
			100,000	
Object position in Z-axis	*hasPosZ*	Object	*double*	0
			100,000	

**Table 12.** Confusion matrix for high-level activities taking as input the sub-activities detected in the first stage tracking system.

Making cereal	1										
Taking medicine		1									
Stacking objects	0.08		0.59	0.33							
Unstacking objects				1							
Microwaving					0.59			0.33			0.08
Picking objects (Bending)			0.25			0.67				0.08	
Cleaning objects							1				
Takeout food							0.08	0.83		0.08	
Arranging objects			0.25	0.08		0.08			0.59		
Eating meal										0.92	0.08
	Making cereal	Taking medicine	Stacking objects	Unstacking objects	Microwaving	Picking objects (Bending)	Cleaning objects	Takeout food	Arranging objects	Eating meal	Null

**Table 13.** Confusion matrix for high-level activities taking as input the sub-activities 100% perfectly labeled from the CAD-120 dataset (ideal condition).

Making cereal	1										
Taking medicine		1									
Stacking objects	0.08		0.59	0.33							
Unstacking objects				0.92					0.08		
Microwaving					0.83			0.17			
Picking objects (Bending)						1					
Cleaning objects							1				
Takeout food								1			
Arranging objects			0.08			0.25			0.67		
Eating Meal										1	
	Making cereal	Taking medicine	Stacking objects	Unstacking objects	Microwaving	Picking objects (Bending)	Cleaning objects	Takeout food	Arranging objects	Eating meal	Null

**Table 14.** Comparison of our approach with the dataset's algorithm.

**Method**	**Sub-Activities input**	**Precision**	**Recall**	**Accuracy**
Koppula *et al.* [27]	Full model with tracking	78.6 ± 4.1	78.3 ± 4.9	79.0 ± 4.7
Ours	Naive approach without heuristic filters	64.1 ± 7.8	75.98 ± 13.53	61.36 ± 6.93
Ours	Real situation (predicted from 1st module-detection system)	84.1 ± 2.3	97.4 ± 0.66	82.9 ± 0.92
Ours	Ideal situation (labeled, 100% acc.)	90.8 ± 1.31	98.1 ± 1.25	91.07 ± 1.28

**Table 15.** Confusion matrix for high-level activities using a naive approach: without applying heuristic filters for the CAD-120 dataset.

Making cereal	0.875			0.125							
Taking medicine		0.875									0.125
Stacking objects			0.167	0.75							0.083
Unstacking objects				1							
Microwaving	0.83				0.583			0.083			0.25
picking objects (Bending)					0.083	0.833			0.83		
Cleaning objects							1				
Takeout food		0.083			0.583		0.167	0.167			
Arranging objects					0.167	0.5			0.083		0.25
Eating Meal					0.083					0.667	0.25
	Making cereal	Taking medicine	Stacking objects	Unstacking objects	Microwaving	Picking objects (Bending)	Cleaning objects	Takeout food	Arranging objects	Eating meal	Null

**Table 16.** Average recognition times (in milliseconds) per high-level activity.

**Activity Recognition Time**	**Average Time**	**Standard Deviation Time**
Making cereal	1,025.94	281.95
Taking medicine	212.9	38.01
Stacking objects	960.12	337.02
Unstacking objects	984.93	283.63
Microwaving food	400.15	229.32
Picking object (Bending)	234.13	301.46
Cleaning objects	480.5	197.79
Taking out food	333.84	249.54
Arranging objects	236.48	253.67
Having meal	733.78	224.85

**Average**	560.28	239.724

## References

[1] Casas R., Blasco Marín R., Robinet A., Delgado A.R., Yarza A.R., Mcginn J., Picking R., Grout V. (2008). User Modeling in Ambient Intelligence for Elderly and Disabled People.

[2] Chen L., Nugent C., Mulvenna M., Finlay D., Hong X., McClean S., Millard P., El-Darzi E., Nugent C. (2009). Semantic Smart Homes: Towards Knowledge Rich Assisted Living Environments. Intelligent Patient Management.

[3] Chen L., Nugent C.D. (2009). Ontology-based activity recognition in intelligent pervasive environments. Int. J. Web Inf. Syst..

[4] Díaz Rodríguez N., Cuéllar M.P., Lilius J., Delgado Calvo-Flores M. (2014). A Survey on Ontologies for Human Behavior Recognition. ACM Comput. Surv..

[5] Díaz Rodríguez N., Cuéllar M.P., Lilius J., Delgado Calvo-Flores M. (2014). A fuzzy ontology for semantic modeling and recognition of human behavior. Knowl. Based Syst..

[6] Baumgartner N., Retschitzegger W. A survey of upper ontologies for situation awareness.

[7] Riboni D., Bettini C. (2011). OWL 2 modeling and reasoning with complex human activities. Pervasive Mob. Comput..

[8] Bettini C., Brdiczka O., Henricksen K., Indulska J., Nicklas D., Ranganathan A., Riboni D. (2010). A survey of context modeling and reasoning techniques. Pervasive Mob. Comput..

[9] Saleemi M., Díaz Rodríguez N., Lilius J., Porres I., Balandin S., Koucheryavi Y., Hu H. (2011). A Framework for Context-Aware Applications for Smart Spaces.

[10] Saleemi M.M., Díaz Rodríguez N., Suenson E., Lilius J., Porres I. (2012). Ontology Driven Smart Space Application Development. Semant Interoper. Issues Solut. Chall..

[11] Baldauf M., Dustdar S., Rosenberg F. (2007). A Survey on Context-Aware Systems. Int. J. Ad. Hoc. Ubiq. Co..

[12] Kowalski R., Sergot M. (1986). A logic-based calculus of events. New Gen. Comput..

[13] Doctor F., Hagras H., Callaghan V. (2005). A fuzzy embedded agent-based approach for realizing ambient intelligence in intelligent inhabited environments. IEEE Trans. Syst. Man Cybern. Part A.

[14] Hagras H., Callaghan V., Colley M., Clarke G., Pounds-Cornish A., Duman H. (2004). Creating an ambient-intelligence environment using embedded agents. IEEE Intell. Syst..

[15] Díaz Rodríguez N., Lilius J., P. Cuéllar M., delgado Calvo-Flores M., Pedrycz W., Reformat M.Z. (2013). An Approach to Improve Semantics in Smart Spaces Using Reactive Fuzzy Rules. IFSA World Congress - NAFIPS Annual Meeting (International Fuzzy Systems Association/North American Fuzzy Information Processing Society).

[16] Pérez I., Wikström R., Mezei J., Carlsson C., Herrera-Viedma E. (2013). A new consensus model for group decision making using fuzzy ontology. Soft Comput..

[17] Pakonen A., Tommila T., Hirvonen J. A fuzzy ontology based approach for mobilising industrial plant knowledge.

[18] Aggarwal J., Ryoo M.S. (2011). Human activity analysis: A review. ACM Comput. Surv..

[19] Raheja J.L., Chaudhary A., Singal K. Tracking of fingertips and centers of palm using kinect.

[20] Giles J. (2010). Inside the race to hack the Kinect. New Sci..

[21] Salas J., Tomasi C. (2011). People detection using color and depth images.

[22] Shotton J., Sharp T., Kipman A., Fitzgibbon A., Finocchio M., Blake A., Cook M., Moore R. (2013). Real-time human pose recognition in parts from single depth images. Commun. ACM.

[23] Yang X., Zhang C., Tian Y., Babaguchi N., Aizawa K., Smith J.R., Satoh S., Plagemann T., Hua X.S., Yan R. (2012). Recognizing Actions Using Depth Motion Maps-Based Histograms of Oriented Gradients. ACM Multimedia.

[24] Yang X., Tian Y. (2013). Effective 3D Action Recognition Using EigenJoints. J. Visual Commun. Image Represent..

[25] Yang X., Tian Y. Eigenjoints-based action recognition using naive-bayes-nearest-neighbor.

[26] Xia L., Chen C.C., Aggarwal J.K. View invariant human action recognition using histograms of 3D joints.

[27] Koppula H.S., Gupta R., Saxena A. (2013). Learning Human Activities and Object Affordances from RGB-D Videos. Int. J. Rob. Res..

[28] Gu T., Chen S., Tao X., Lu J. (2010). An unsupervised approach to activity recognition and segmentation based on object-use fingerprints. Data Knowl. Eng..

[29] Borst W.N. (1997). Construction of Engineering Ontologies for Knowledge Sharing and Reuse. Ph.D. Thesis.

[30] Bobillo F. (2008). Managing Vagueness in Ontologies. Ph.D. Thesis.

[31] Chen L.L., Biswas J. Tutorial: An introduction to ontology-based activity recognition.

[32] Baader F., Horrocks I., Sattler U., van Harmelen F., Lifschitz V., Porter B. (2007). Description Logics. Handbook of Knowledge Representation.

[33] Gómez-Romero J., Patricio M.A., García J., Molina J.M. (2011). Ontology-based context representation and reasoning for object tracking and scene interpretation in video. Expert Syst. Appl..

[34] Yaouanc J.M., Poli J.P., Castano S., Vassiliadis P., Lakshmanan L., Lee M. (2012). A Fuzzy Spatio-Temporal-Based Approach for Activity Recognition. Advances in Conceptual Modeling.

[35] Ye J., Stevenson G., Dobson S. (2014). KCAR: A knowledge-driven approach for concurrent activity recognition. Pervasive Mob. Comput..

[36] Chen L., Nugent C.D., Wang H. (2012). A Knowledge-Driven Approach to Activity Recognition in Smart Homes. IEEE Trans. Knowl. Data Eng..

[37] Saguna S., Zaslavsky A., Chakraborty D. (2013). Complex Activity Recognition Using Context-Driven Activity Theory and Activity Signatures. ACM Trans. Comput. Hum. Interact..

[38] Okeyo G., Chen L., Wang H. (2014). Combining ontological and temporal formalisms for composite activity modeling and recognition in smart homes. Future Gener. Comput. Syst..

[39] Okeyo G., Chen L., Wang H., Sterritt R. (2014). Dynamic sensor data segmentation for real-time knowledge-driven activity recognition. Pervasive Mob. Comput..

[40] Ikizler-Cinbis N., Sclaroff S. (2010). Object, Scene and Actions: Combining Multiple Features for Human Action Recognition.

[41] Lai K., Bo L., Ren X., Fox D. A large-scale hierarchical multi-view rgb-d object dataset.

[42] Keogh E., Chakrabarti K., Pazzani M., Mehrotra S. (2001). Dimensionality Reduction for Fast Similarity Search in Large Time Series Databases. Knowl. Inf. Syst..

[43] Corradini A. Dynamic time warping for off-line recognition of a small gesture vocabulary.

[44] León O., Cuéllar M.P., Delgado M., Borgne Y.L., Bontempi G. Human Activity Recognition Framework in Monitored Environments.

[45] Zadeh L. (1965). Fuzzy Sets. Inf. Control.

[46] Bobillo F., Straccia U. fuzzyDL: An Expressive Fuzzy Description Logic Reasoner.

[47] Bobillo F., Straccia U. (2011). Fuzzy ontology representation using OWL 2. Int. J. Approx. Reason..

[48] Tiberghien T., Mokhtari M., Aloulou H., Biswas J., Cudré-Mauroux P., Heflin J., Sirin E., Tudorache T., Euzenat J., Hauswirth M., Parreira J., Hendler J., Schreiber G., Bernstein A. (2012). Semantic Reasoning in Context-Aware Assistive Environments to Support Ageing with Dementia. The Semantic Web–ISWC (International Semantic Web Conference) 2012.

[49] Ros M., Cuéllar M., Delgado M., Vila A. (2013). Online recognition of human activities and adaptation to habit changes by means of learning automata and fuzzy temporal windows. Inf. Sci..

[50] Riboni D., Bettini C. (2011). COSAR: Hybrid reasoning for context-aware activity recognition. Pers. Ubiquitous Comput..

[51] Poveda-Villalón M., Suárez-Figueroa M.C., García-Castro R., Gómez-Pérez A. (2010). A Context Ontology for Mobile Environments.

[52] Wang X.H., Zhang D., Gu T., Pung H. Ontology Based Context Modeling and Reasoning using OWL.

[53] Hervás R., Bravo J., Fontecha J. (2010). A Context Model Based on Ontological Languages: A Proposal for Information Visualization. J. Univ. Comput. Sci..

[54] Yau S.S., Liu J. (2006). Hierarchical Situation Modeling and Reasoning for Pervasive Computing.

